# Evaluating a training intervention for improving alignment between emergency medical telephone operators and callers: a pilot study of communication behaviours

**DOI:** 10.1186/s13049-021-00917-y

**Published:** 2021-07-31

**Authors:** Jennifer Gerwing, Jon Erik Steen-Hansen, Trond Mjaaland, Bård Fossli Jensen, Olav Eielsen, Owen Matthew Truscott Thomas, Pål Gulbrandsen

**Affiliations:** 1 Health Services Research Unit, Akershus University Hospital, Sykehusveien 25, 1478 Lørenskog, Norway; 2 Division of Prehospital Care, Vestfold Hospital Trust, Box 2168, NO-3103 Tønsberg, Norway; 3 Somsagt AS, Forskningsparken / Oslo Science Park, Gaustadalleen 21, 0349 Oslo, Norway; 4 NorSahel, Fjonevegen 72, 3855 Treungen, Norway; 5 Institute of Clinical Medicine, University of Oslo, PO box 1171, Blindern, 0318 Oslo, Norway

**Keywords:** Emergency medical services dispatch, Emergency medical communication Centre, Emergency calls, Communication, Communication training, Empathy, Norway

## Abstract

**Background:**

Calls to emergency medical lines are an essential component in the chain of survival. Operators make critical decisions based on information they elicit from callers. Although smooth cooperation is necessary, the field lacks evidence-based guidelines for how to achieve it while adhering to strict parameters of index-driven questioning. We aimed to evaluate the effect of a training intervention for emergency medical operators at a call centre in Tønsberg, Norway. The course was designed to enhance operators’ communication skills for smoothing cooperation with callers.

**Methods:**

Calls were analyzed using inductively developed coding based on the course rationale and content. To evaluate whether the course generated consolidated behavioral change in everyday practice, the independent analyst evaluated 32 calls, selected randomly from eight operators, two calls before and two after course completion. To measure whether skill attainment delayed decision making, we compared the time to the first decision logged by intervention operators to eight control operators. Analysis included 3034 calls: 1375 to intervention operators (T1 = 815; T2 = 560) and 1659 to control operators (T1 = 683; T2 = 976).

**Results:**

Operators demonstrated improved behaviours on how they *greeted the caller* (*p* < .001), *acknowledged* the caller (*p* < .001), and *displayed empathy* (*p* = 0.015). No change was found in the use of *open-ended questions* and *agreeing* with the caller. Contrary to expectations, operators who took the course logged first decisions more quickly than the control group (*p* < .001).

**Conclusions:**

This pilot study demonstrated that the training intervention generated behavioural change in these operators, providing justification for scaling up the intervention.

**Supplementary Information:**

The online version contains supplementary material available at 10.1186/s13049-021-00917-y.

## Introduction

The dispatch of prehospital emergency medical resources is organized differently worldwide. In Scandinavia, Denmark and Norway have dedicated Emergency Medical Communication Centers (EMCC - phone 113/112), which are hospital organized, an integrated part of the health care system, and staffed with health care professionals. Norway is unique in that health care professionals answer calls to 113 directly, whereas in Denmark, police officers answer calls to 112 and then forward them to the EMCC after a decision-making process. Since EMCCs were established across Norway in the early 1990s, the main focus in operator training has been the national interrogation algorithms and flow charts for telephone triage, dispatch, and telephone instruction in life-saving first aid (Norsk indeks for medisinsk nødhjelp, *trans. Norwegian Index for Emergency Medical Assistance*) [1], which has been shown to aid EMCC operators distinguish between patients of high vs. low acuity [2]. This “Index-type” interrogation and commanding style may constitute more of an obstacle than an aid to the person in need of emergency medical interventions; adhering to the algorithm while displaying empathy to the caller constitutes a central challenge to practice.

## Background

Calls to medical emergency lines constitute an essential component in the chain of survival, yet they have been characterized as a ‘black box’ in research on prehospital care [3]. In these calls, operators rely on what they can elicit from callers to visualize the current and evolving situation [4], which they combine with professional guidelines and available resources to make critical decisions [5]. While operators achieve high levels of accuracy with the most urgent cases, their decision accuracy falters when the patient’s situation appears ambiguous or vague [5, 6]. In this dynamic, interactional arena, operators acknowledge the essential role of smooth cooperation with the caller [7]. However, the lack of common ground between caller and operator challenges such cooperation. Operators and callers are in different worlds [7], in terms of their roles, responsibilities, knowledge, emotions, and familiarity with the situation (see Table 1).

**Table 1:** Areas of common ground lacking between citizen callers and EMCC operators

	Lay person caller	EMCC Operator
*Role*	Someone who needs help	A health care professional who provides help
*Epistemic domain*	Immediate access to self (or other person), unfolding situation, history	Medical, triage routine, health care system, potential patient records
*Responsibility*	Moral (whether calling for self or other)	Professional
*Emotional proximity*	Potentially distressing, frightening	Distant, professional
*Familiarity with situation*	Likely low, extraordinary situation	High, everyday, routine work

Previous research has revealed conversational effects and negative outcomes related to misalignment in expectations [8], knowledge about the emergency system [9], and medical matters [10]. For example, operators recognize cardiac arrest later when they pursue a line of questioning based on callers’ presumptive diagnostic reports (e.g., heart attack, stroke) rather than obtaining descriptions of symptoms (consciousness and breathing) [10]. Callers may not recognize diagnostically important symptoms (e.g., agonal breathing) and may consequently produce unintentionally misleading answers to questions (e.g., “is he breathing?”) [3]. Unintentionally misleading answers are not limited to medical knowledge; for example, one study showed that callers reported that the patient was sitting, when CCTV footage clearly revealed that bystanders were holding the unconscious patient in a seated position [11]. Mismatches in emotional states present a particular challenge. Operators who address callers’ emotional clues and displays of urgency maintain effective communication, thereby obtaining the information they need [12]. In contrast, not aligning with a caller’s emotional state can generate conflict that delays critical care [13]. Operators report finding callers’ levels of distress a particular obstacle for their efforts to triage [5]; indeed, callers with high levels of emotional distress obstruct recognition of cardiac arrest [7].

Despite the need for cooperation, operators have few evidence-based guidelines on how to achieve it while following the strict parameters of index-driven questioning. Analysis of communication and intervention studies have existed in separate silos, with linguistically-oriented studies revealing important concrete effects of subtle differences in communication behavior in actual calls without any training interventions (e.g., [3, 14, 15]), and intervention studies focusing on formal verbatim scripts applied in simulations (e.g., [16]). The field lacks a description and evaluation of a comprehensive, trainable suite of communication strategies for increasing alignment between caller and operator, measured in routinely recorded calls.

This study aimed to evaluate a training intervention designed to enhance EMCC operators’ functional communication skills for aligning with callers regarding their different roles, knowledge, responsibility, and emotional involvement. A further aim was to check whether the attainment of skills increased operators’ decision-making time.

## Method

### Research design

This is a pilot study of an evaluation of a training course for EMCC operators. To evaluate whether the course resulted in sustained and consolidated behavioral change in everyday practice, an independent analyst (JG) evaluated a random selection of calls before and after the course. To discern whether taking the course increased decision-making time, we measured how long it took for operators to log their first decision (i.e., take their first “action”, such as *dispatching* Emergency Ambulance, Emergency vehicle with anesthetist, Emergency vehicle with GP, Air Ambulance, Police, Fire brigade, *transferring* the call to emergency primary health care, GP's office, *providing telephone advice* when triage indicates that the patient does not need medical treatment in the next 24 h).

### Participants

The pilot study was conducted at EMCC in Tønsberg, situated at Vestfold Hospital Trust (Norway), covering a population of approximately 420,000 people. The intervention group included 8 EMCC operators who were recruited from the approximately 30 operators working at the centre. The call takers are usually registered nurses, trained for the role as an EMCC operator through a program consisting of 140 h of theory and 280 h of practical training. After being guided by an experienced colleague for some time, call takers begin increasingly independent work. After passing an approval test, they are allowed to work fully independently. Operators who applied to take the course completed a competency and motivational interview, those who scored highest were accepted. A control group of 8 operators were included in the time-to-action analysis. These operators had expressed interest in training, which they had not yet received, although they had completed a one-day orientation of the basic principles in communication psychology. The characteristics of the two groups were the same: Both acted as medical operators (i.e., 113 call takers), as opposed to resource coordinators (who assign and manage resources to events decided by the medical operator), and all were employed at the EMCC throughout the period from February 2019 through September 2019. Additional File 1 provides supplementary data on the emergency call operators.

### Intervention

The duration of the course was open-ended, with training continuing until the teachers (TM and BFJ) evaluated the operators’ attainment of skills sufficient for mastering in practice without assistance. The course included 3 days of group education and hours individually working with logged (i.e., recorded) calls. To measure the level of skill attainment and the theoretical understanding of the psychological mechanisms, operators completed a written and oral test. All operators passed. Details regarding all aspects of the training are available upon request.

### Call selection

The EMCC receives enquiries through several channels from professionals and lay people on a variety of events. This project was focused on communication in *calls to 113,* from the *general public* about an *emergency situation.* Thus, calls were screened for relevance using variables that are automatically registered by the system (e.g., type of line, name of calling organization) or manually routinely registered by the operators (e.g., type of enquiry, caller, event). The Medical Director (JESH) screened all calls to these 16 operators, applying inclusion and exclusion criteria (listed in Table 2) from two relevant time periods before (T1) and after (T2) the course, which spanned mid-March 2019 to the end of June 2019. Taking into account large seasonal variations that influence the number and nature of enquiries, T1 was defined as the “last normal month” before training (February 2019) and T2 as the “first normal month after training” (September 2019).

**Table 2:** Inclusion and exclusion criteria for selection of calls

Variable	Included	Excluded
Enquiry	Calls to 113	“Calls” from other lines as Ambulance booking, Web booking, Emergency room, lines from Fire, Police, Radio and administrative telephone lines.
Caller	The general public (Patient, Relatives, Children under the age of 16, Neighbor, Bystander)	Doctor, Health Professionals, Emergency primary health care central, Other EMCC Central, Fire Department, Police, Airline, Paramedics.
Event	Somatic disease, Obstetrics/ Maternity Care, Psychiatry / psychosocial, Overdose addict, Intoxication Traffic accident, Fire, Other type of accident, Violence - exposed to Violence - self-inflicted, Medical emergency – other, Obvious signs of death, Search and rescue Preparedness v/dangerous situation Harassment, Emergency telephone for the Police, Emergency telephone for the fire brigade	Transfer, Homerun, To doctor/outpatient clinic, Other booking Other inquiry –no need, Hospital internal emergency, Other internal incident, Emergency response moved (tactical displacement of empty ambulance to maintain dispersed emergency response), False message, error ring, Technical error ring Shredded.

For the communication analysis, we used only calls to the 8 intervention operators. Data were 32 calls randomly selected from the above, 4 per operator, 2 from T1 and 2 from T2. This additional random selection procedure is described in detail in Additional File 1.

**Fig. 1:**
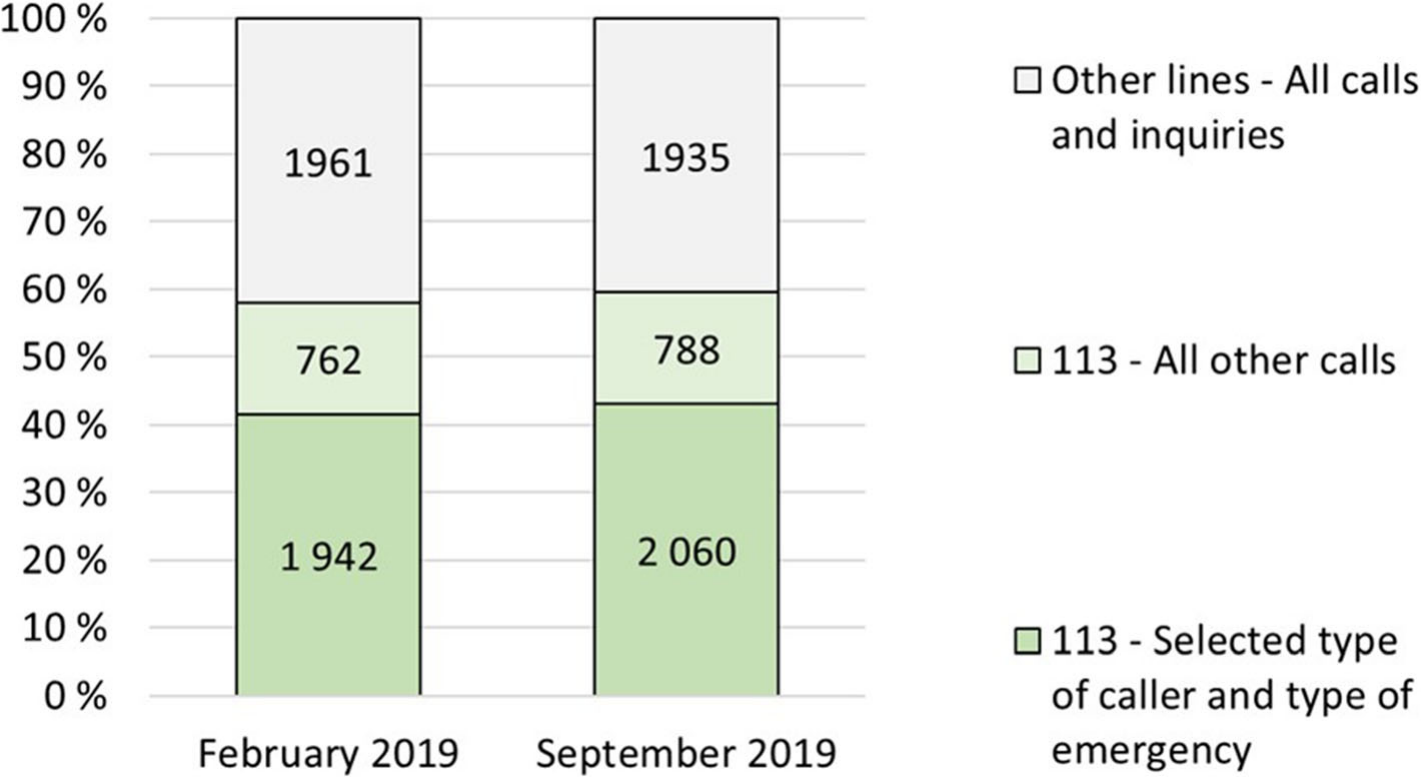
All emergency calls and other inquiries (all call-takers) to the EMCC during the study periods

For the time-to-action analysis, the data were all calls screened for relevancy to the 8 intervention and 8 control operators from the two time periods. This selection process yielded 3034 calls: 1375 to the intervention operators (T1 = 815; T2 = 560) and 1659 to the control operators (T1 = 683; T2 = 976). Figure 1 provides the distribution of all inquiries to all call-takers (the study group, the control group and the remaining call-taker operators) of the EMCC in the two time periods, and Additional File 1 provides a detailed account of the 8 intervention and 8 control operators, including their experience and activities in the two time periods.

## Analysis

### Communication analysis: definitions and procedures

The communication analyst (JG) was blind as to which calls were T1 or T2. The method of analysis was microanalysis of face-to-face dialogue [17], adapted to the telephone setting. JG used transcripts with audio files (to hear tone of voice, pauses, overlapping speech), using ELAN [18, 19] to annotate selections for repeated listening when required. JG developed the analysis inductively, based on (1) the trainers’ descriptions of their rationale and overarching teaching aims, (2) specific course content and examples, and (3) linguistic background knowledge. The behaviours of interest are presented briefly in Table 3 and in more detail in Additional File 2.

**Table 3:** Communication behaviours analyzed, rationale, and definition

Derived from course description		Product of analysis of calls
Behavior	Rationale	Brief definition for positive evaluation
*Greeting*	decreases uncertainty at the outset of the call and defines the operator’s role	the number of the centre (113), the operator’s name, and (if applicable) that they are a nurse
*Open-ended questions*	gives callers maximum autonomy, accountability, and opportunity to describe what is within their epistemic domain (i.e., their situation)	request for information that requires a substantive answer from the caller (i.e., more than yes, no, or choice from a closed list of options)
*Acknowledging the caller*	signals that the caller’s actions reflect fulfilled moral or ethical responsibility	statements that thank or compliment the caller’s contributions and actions
*Expressing empathy*	bridges emotional distance, allowing the caller to feel the operator’s compassion	expressions or utterances that *share*, *match*, or *directly address* the caller’s observable positive or negative state
*Agreeing with the caller (avoiding disagreements)*	offers callers the sense that the operator has confidence in their ability to observe and evaluate the situation	the lack of a negative response to caller’s opinions/evaluations that would normally be within the operator’s epistemic domain

JG used ten of the 32 calls to develop a detailed coding manual (which includes all operational definitions, procedures, and multiple examples) and then applied the coding to the remaining 22 calls. She entered all analytical decisions for each transcript into excel (one worksheet per call, one utterance per row), noting analytical decisions for each utterance, checking for consistent decision making, and discussing difficult coding decisions with PG and interaction analysts from our research group. (The coding manual is available by request from JG.) During coding, no contact was made with the course instructors (BFJ and TM).

### Time to action analysis: definition and procedure

In the case of all answered enquiries where contact with a caller to EMCC is established, the EMCC operator always logs at least one decision on “Action”. Often there are several actions, automatically recorded in chronological order. The first action, therefore, is defined as a registration of the first decision that an EMCC operator logs. The selected indicator for time-to-action in this study is: *the interval, in seconds, from when the caller makes contact with the operator until the operator logs the first action*.

### Statistical procedures

For the trained communicative behaviours, the statistical task was to compare various behavioural features of operators of emergency calls before and after training, with a view to understanding whether the training had any effect on the behaviour. Each operator was analysed twice before the training and twice after, suggesting that a multi-level analysis would be appropriate. The statistical task was performed using the lmer package for linear mixed models in R [20], using the function “glmer”, allowing for the use of different likelihoods for each type of data. The binary variable indicating whether training had occurred or not was used as a covariate, and the label associated with each operator was used to define the multilevel structure, assigning each operator their own intercept value in the linear model. The model coefficient associated with the training was used to evaluate the influence of the intervention: a value of zero would correspond with no effect, and high likelihood of coefficient values away from zero is evidence for the intervention influencing the outcome. For open questions, a generalised linear mixed model with a binomial likelihood was used to model the proportion of open questions, with each proportion weighted by the number of total questions used to calculate the proportion. For measuring acknowledging and empathic behaviours, we use an integer count, so a Poisson likelihood was used for the general linear model with mixed effects.

For time to action we used a hierarchical generalized linear model with a Gamma likelihood, using the operator IDs to define the random effects and the intervention status as the fixed effect. This statistic uses all of the data, including the control group, to determine the effect of the intervention.

## Results

Additional File 1 includes a description of the 32 sampled calls.

### Trained communication behaviours

Table 4 presents the scoring and descriptive statistics for each variable and the results of significance testing.

**Table 4:** Scoring and descriptive statistics for analysis of communication behaviours

Behavior	Scoring for each call	M (SD) Range	
		Pre-training	Post-training
Greeting**	Dichotomous: 1 = greeting fulfils criteria; 0 = greeting does not fulfill criteria	0.00 (0.00) 0.00-0.00	1.00 (0.00) 1.00-1.00
*Open-ended questions*	Proportion: The number of open-ended questions over all questions	0.42 (0.21) 0.07-1.00	0.46 (0.23) 0.12-1.00
Acknowledging the caller**	Frequency: The number of utterances during which the operator acknowledges the caller	0.10 (0.30) 0.00-1.00	1.60 (2.00) 0.00-6.00
Expressing empathy*	Frequency: The number of utterances during which the operator expresses empathy to the caller	1.50 (2.06) 0.00-6.00	2.80 (3.33) 0.00-9.00
*Agreeing with the caller (avoiding disagreements)*	Proportion: The number of not-negative responses over opportunities	0.97 0.80-1.00	1.00 1.00-1.00

*p = 0.015; ** *p* < .001

#### Greeting the caller

The training had a very strong effect on caller greeting, with none of the operators using the correct greeting before the training and all but one of the data points after the training (in which the operator’s greeting was not applicable) featuring the correct greeting. A Fisher’s exact test on a contingency table of the intervention and the greeting used returned a very small *p*-value (*p* < .001).

#### Asking open-ended questions

The training program did not have a clear effect on the proportion of open questions asked by the operator. The generalised linear model coefficient associated with the influence of the intervention had a value of − 0.01 with a 95% confidence interval [− 0.42, 0.39] and associated p-value of 0.945, suggesting little to no effect.

#### Acknowledging the caller

The effect of training was found to be highly influential on the degree of acknowledgement of the operators. The generalised linear model coefficient associated with the intervention had a value of 2.56, with an associated 95% confidence interval of [1.13, 4.00], and associated *p*-value (*p* < .001), suggesting a very strong effect of the intervention.

#### Expressing empathy to the caller

The training intervention was found to produce a strong effect on the empathy behaviour of the operators, with the generalised linear model coefficient associated with the intervention taking a value of 0.61 and 95% confidence interval [0.12, 1.09], corresponding to a *p*-value of 0.015.

#### Agreeing with the caller

All but one of the proportions described in the data set were either 1 or n/a, suggesting that the operators responded positively with the caller at every opportunity except one (both at T1 and T2). As such, we expected the training to have little effect on the behaviour outcome, in a nearly exact opposite situation to the greeting variable. A Fisher’s exact test with no multi-level structure, performed on a contingency table defined over the intervention and the agreement data, demonstrated little to no effect of the training on the outcome (*p* = 0.3333).

### Time to action

The intervention operators made their first decisions more quickly after the course, compared to the control operators. For the intervention operators, the average of time-to-action at T1 was 169 s (SD = 413) and T2 was 141 s (SD = 149). For the control operators, at T1, the average was 193 s (SD = 224) and at T2 was 178 s (SD = 323). The generalised linear mixed model used a Gamma likelihood for the data, and included random effects associated with each operator, and fixed effects associated with the different time points and the effect of the intervention at the second time point. A strong effect was found associated with the coefficient describing the effect of the intervention, with a coefficient value of − 0.22 and corresponding 95% confidence interval of [− 0.31, − 0.13], corresponding to a very small *p*-value (*p* < .001).

## Discussion

The training course improved EMCC operators’ greeting behaviours, the frequency with which they acknowledged and thanked callers for their cooperation, and the frequency with which they expressed empathy by briefly affiliating, accommodating, or directly addressing the caller’s emotional needs. It did not improve the extent to which the operators used open-ended questions (about half open-ended questions before and after the course). Finally, both before and after the course, the operators did not take time to disagree with callers in response to their proposals that were within the operator’s professional domain (e.g., diagnosing the patient, triaging the situation). There was a concern that training the operators to use communication behaviours focused on psychological aspects of the conversation might increase the time that the calls required. On the contrary, results demonstrated the opposite: operators logged the first decision more quickly after the course.

In EMCC’s in general, the availability of automatically recorded calls provides a rare opportunity to peer into the ‘black box’ of pre-hospital care. Retrospective access allowed us to compare EMCC operators’ communication skills before the course was announced to 2 months after course completion. Producing robust and sustained behavioural change is a challenge for clinical communication training interventions: In a so-called “skills to performance gap”, practitioners tend to demonstrate better skills during (or immediately following) an intervention compared during the demands of unobserved everyday practice (e.g., [21, 22] p. 134-135). The skills to performance gap was not apparent with these operators, as shown by the randomly selected calls chosen from operators’ daily work after course completion (i.e., not calls when they were aware that their performance was being evaluated).

The training intervention had qualities consistent with a recent systematic review, which reported that the most effective methods for training about empathy and compassion involve a didactic approach in combination with multiple practice opportunities and tailored feedback [23]. This review also supported teaching skills specifically focused on responding to opportunities for compassion and acknowledgement, although there is no agreed-upon method for measuring whether practitioners have attained these skills. The coding developed here constitutes a step in that direction, as it is a precise, quantifiable method with results that represent aggregated scores, traceable to precise moments in each call.

### Strengths

This study took a unique approach, measuring communication behaviours in parallel with indexed evaluation protocols. It was a pilot test intended to indicate whether the course was effective under the best conditions (e.g., a small set of highly-motivated operators). As such, the study constitutes an essential first step for scaling up to a randomized controlled trial. A study of interviews with medical dispatchers in Denmark indicated that one of the most “potent” modifiable factors in handling emergency calls was continuous professional development, including feedback on recordings of calls for reflection on competence [24]; the behaviours taught and operationalized here provide indicators for what could be the focus of feedback and considered competent regarding how to align expectations, knowledge, and emotions.

### Limitations

Whether the intervention would be as effective with less motivated operators is an open question. The course required extensive time and multiple training methods, and some efforts could be taken to test which aspects of the course are essential for producing robust behavioural change. Numerous studies have pointed to specific difficulties in calls that involve linguistic misalignment (e.g., [13, 25–27]), which this study did not address. How operators can display empathy, acknowledgement, and agreement when there is a language barrier is worthy of further investigation.

## Conclusions

When EMCC operators and callers struggle to cooperate (due to emotions, confusion, and conflict), operators’ critical decision-making abilities are disrupted, which can lead to pre-hospital delays or over- or under-use of resources. Operators can learn techniques for reducing uncertainty, showing appreciation (e.g., for good information), and offering signs of understanding. Index-driven questioning need not be abandoned, as these techniques were easily integrated into routines, demonstrating that EMCC operators can balance strict guidelines with signs of compassion. In this pilot study, such psychological techniques reduced rather than increased decision-making time.

## Supplementary Information

**Additional file 1.**

**Additional file 2.**

## Data Availability

The datasets generated and/or analysed during the current study are not publicly available due to confidentiality and anonymity for both callers and operators, who could be identifiable from voice recordings or details available in transcripts. Anonymized transcripts could be available from the corresponding author on reasonable request.

## References

[1] Norsk indeks for medisinsk nødhjelp (2018). Oslo, Norwegian National Advisory Unit on Prehospital Emergency Medicine (NAKOS).

[2] Grusd E, Kramer-Johansen J (2016). Does the Norwegian emergency medical dispatch classification as non-urgent predict no need for pre-hospital medical treatment? An observational study. Scand J Trauma Resusc Emerg Med.

[3] Riou M, Ball S, Williams TA, Whiteside A, Cameron P, Fatovich DM (2018). ‘She’s sort of breathing’: what linguistic factors determine call-taker recognition of agonal breathing in emergency calls for cardiac arrest?. Resuscitation..

[4] Edwards B (1998). Seeing is believing--picture building: a key component of telephone triage. J Clin Nurs.

[5] Leonardsen AC, Ramsdal H, Olasveengen TM, Steen-Hansen JE, Westmark F, Hansen AE (2019). Exploring individual and work organizational peculiarities of working in emergency medical communication centers in Norway-a qualitative study. BMC Health Serv Res.

[6] Leprohon J, Patel VL (1995). Decision-making strategies for telephone triage in emergency medical services. Med Decis Mak.

[7] Hardeland C, Sunde K, Ramsdal H, Hebbert SR, Soilammi L, Westmark F (2016). Factors impacting upon timely and adequate allocation of prehospital medical assistance and resources to cardiac arrest patients. Resuscitation..

[8] Tracy K (1997). Interactional trouble in emergency service requests: a problem of frames. Res Lang Soc Interact.

[9] Paoletti I (2012). The issue of conversationally constituted context and localization problems in emergency calls. Text Talk.

[10] Stangenes SR, Painter IS, Rea TD, Meischke H (2020). Delays in recognition of the need for telephone-assisted CPR due to caller descriptions of chief complaint. Resuscitation..

[11] Linderoth G, Hallas P, Lippert FK, Wibrandt I, Loumann S, Møller TP, Østergaard D (2015). Challenges in out-of-hospital cardiac arrest–a study combining closed-circuit television (CCTV) and medical emergency calls. Resuscitation..

[12] Paoletti I (2012). Operators managing callers’ sense of urgency in calls to the medical emergency number. Pragmatics..

[13] Svennevig J (2012). On being heard in emergency calls. The development of hostility in a fatal emergency call. J Pragmat.

[14] Riou M, Ball S, Williams TA, Whiteside A, O’Halloran KL, Bray J (2017). ‘Tell me exactly what’s happened’: when linguistic choices affect the efficiency of emergency calls for cardiac arrest. Resuscitation..

[15] Riou M, Ball S, Whiteside A, Bray J, Perkins GD, Smith K, O’Halloran KL (2018). ‘We’re going to do CPR’: a linguistic study of the words used to initiate dispatcher-assisted CPR and their association with caller agreement. Resuscitation.

[16] Painter I, Chavez DE, Ike BR, Yip MP, Tu SP, Bradley SM (2014). Changes to DA-CPR instructions: can we reduce time to first compression and improve quality of bystander CPR?. Resuscitation..

[17] Bavelas JB, Gerwing J, Healing S, Tomori C, Van Lear CA, Canary DJ (2016). Microanalysis of face-to-face dialogue: an inductive approach. Researching communication interaction behavior: a sourcebook of methods and measures.

[18] ELAN (Version 6.0) [Computer software]. (2020). Nijmegen: Max Planck Institute for Psycholinguistics, The Language Archive. Retrieved from https://archive.mpi.nl/tla/elan.

[19] Wittenburg P, Brugman H, Russel A, Klassmann A, Sloetjes H (2006). ELAN: a professional framework for multimodality research. Proceedings of LREC 2006, Fifth International Conference on Language Resources and Evaluation.

[20] Kuznetsova A, Brockhoff PB, Christensen RHB (2017). lmerTest Package: tests in linear mixed effects models. J Stat Softw.

[21] Schwartz A, Weiner SJ, Harris IB, Binns-Calvey A (2010). An educational intervention for contextualizing patient care and medical students' abilities to probe for contextual issues in simulated patients. Jama..

[22] Weiner SJ, Schwartz A (2016). Listening for what matters: Avoiding contextual errors in health care.

[23] Patel S, Pelletier-Bui A, Smith S, Roberts MB, Kilgannon H, Trzeciak S (2019). Curricula for empathy and compassion training in medical education: a systematic review. PLoS One.

[24] Møller TP, Jensen HG, Viereck S, Lippert F, Østergaaard D (2021). Medical dispatchers’ perception of the interaction with the caller during emergency calls-a qualitative study. Scand J Trauma Resusc Emerg Med.

[25] Osvaldsson K, Persson-Thunqvist D, Cromdal J (2013). Comprehension checks, clarifications, and corrections in an emergency call with a nonnative speaker of Swedish. Int J Bilingual.

[26] Penn C, Watermeyer J, Nattrass R (2017). Managing language mismatches in emergency calls. J Health Psychol.

[27] Gerwing J, Indseth T (2010). Communication with non-native callers in medical emergency calls: Recommendations for AMK operators and leadership.

