# Supplementary material for: Evaluating a training intervention for improving alignment between emergency medical telephone operators and callers: a pilot study of communication behaviours
Source: Scand J Trauma Resusc Emerg Med. 2021 Jul 31;29:107. doi: 10.1186/s13049-021-00917-y (PMC8325801; doi:10.1186/s13049-021-00917-y)
Supplement: Supplementary file 1 — Additional file 1. [file 13049_2021_917_MOESM1_ESM.docx]

# Additional file 1

## Data on emergency call operators

|  |  | |  | | T1: February 2019 | | | T2: September 2019 | | |  |  |
| --- | --- | --- | --- | --- | --- | --- | --- | --- | --- | --- | --- | --- |
| Intervention operators | Mean Job experience (years) | | Full/Part-time job in percent | | Number of shifts | Working hours | Answered calls | Number of shifts | Working hours | Answered calls | Group number of calls | Group number of working hours |
| A | 3 | | 100 % | | 12 | 112.75 | 161 | 13 | 123 | 168 |  |  |
| B | 8 | | 100 % | | 12 | 142.76 | 51 | 12 | 141.42 | 17 |  |  |
| C | 14 | | 100 % | | 12 | 147 | 162 | 9 | 110.25 | 100 |  |  |
| D | 1 | | 50 % | | 9 | 103.25 | 108 | 9 | 110.25 | 119 |  |  |
| E | 1 | | 100 % | | 8 | 98.00 | 110 | 6 | 73.5 | 60 |  |  |
| F | 8 | | 25 % | | 6 | 52.5 | 36 | 5 | 45 | 63 |  |  |
| G | 2 | | 75 % | | 13 | 113 | 111 | 3 | 22.5 | 12 |  |  |
| H | 7 | | 100 % | | 10 | 118.25 | 76 | 11 | 134.75 | 21 |  |  |
| SUM | | | | | 82 | 887.51 | 815 | 68 | 760.67 | 560 | 1375 | 1648 |
| Mean | 5.5 | | 81 % | |  |  |  |  |  |  |  |  |
| Control group operators | Mean Job experience (years) | | Full/Part-time job in percent | | Number of shifts | Working hours | Answered calls | Number of shifts | Working hours | Answered calls |  |  |
| I | 3 | | 100 % | | 7 | 87.5 | 95 | 11 | 134.75 | 157 |  |  |
| J | 2 | | 50 % | | 4 | 44.75 | 61 | 7 | 85.75 | 103 |  |  |
| K | 10 | | 100 % | | 11 | 134.75 | 137 | 14 | 156.5 | 183 |  |  |
| L | 1 | | 75 % | | 6 | 73.5 | 89 | 6 | 73.5 | 100 |  |  |
| M | 1 | | 75 % | | 12 | 110.5 | 17 | 14 | 165.9 | 75 |  |  |
| N | 11 | | 100 % | | 11 | 126.75 | 50 | 11 | 113.5 | 17 |  |  |
| O | 14 | | 100 % | | 12 | 147 | 190 | 12 | 147 | 160 |  |  |
| P | 8 | | 100 % | | 3 | 36.75 | 44 | 11 | 134.75 | 181 |  |  |
| SUM | | | | | 66 | 761.5 | 683 | 86 | 1011.65 | 976 | 1659 | 1773 |
| Mean | | 6,3 | | 88% |  |  |  |  |  |  |  |  |

### The 32 sampled calls

Random selection: One Co-worker (Trond Thoresen) initially configured a report in Crystal Reports, which extracted and exported the necessary relevant data for this study and study period (event number, call taker ID, incoming line, type of caller, medical triage criteria’s, Date/time points, type of action etc.) from the AMIS database to an excel sheet. Co-author JESH isolated all event numbers from the study period with following inclusion criteria: (1) Incoming line = 113, (2) Caller type “General public” (Witness, neighbor, relative, patient, children below 16 years), (3) Call answered by call takers defined as “study group”.

A second co-worker (Peter Fiskerstrand) used Excel to randomize four event numbers (two before and two after the course) for each of the call takers in the study group. The sound files from these randomized events were located in the audio log system (NICE Inform) using the exact time in dd-mmm-yyyy-hh-mm-ss and incoming 113-line. Co-author JESH controlled the soundfiles by listening for accuracy of caller type coding (which had been manually registered by the call taker during the call). One of the original 32 soundfiles had a false coded caller type and had to be replaced by randomizing one more event number for the same call taker.

**Description of 32 randomly selected calls**

Twelve callers were calling for themselves, the remaining 20 callers were calling on behalf of someone else: spouse (7), other family member (4), their child/baby (2), a friend (2), a neighbor (1), co-worker (1). Three callers were bystanders (at a bus stop, a bar, and on the highway).

In the 32 calls, only five callers explicitly requested an ambulance; the operators decided to send one for 22 calls (including those five). The operators’ other final decisions were: connecting the caller directly to Emergency primary health care) (3), directly to a doctor (2), arranging to call for a specialist at the hospital to contact the caller (1), suggesting the caller telephone Emergency primary health care central (telephone number 116117) (1), and arranging to call ahead to Emergency primary health care office , where the caller was heading already (1). Two calls resolved without the operator sending or arranging any resources.
