# Supplementary material for: Evaluating a training intervention for improving alignment between emergency medical telephone operators and callers: a pilot study of communication behaviours
Source: Scand J Trauma Resusc Emerg Med. 2021 Jul 31;29:107. doi: 10.1186/s13049-021-00917-y (PMC8325801; doi:10.1186/s13049-021-00917-y)
Supplement: Supplementary file 2 — Additional file 2. [file 13049_2021_917_MOESM2_ESM.docx]

# Additional file 2

# Definitions and examples of the communication behaviour measures

#### Greeting the caller

The greeting was the first utterance in which the operator addressed the caller. The operators were taught the following formula: say the number the caller dialed (“113”), say “nurse” if applicable, and say “(name)”.

#### Asking open-ended questions

First, the analyst identified all utterances during which the operator requested information from the caller, defined as utterances in which the operator invited the caller to provide new information. These could take the grammatical form of a question (e.g., “What can I help you with?”), a polite imperative (“Can you tell me where you need help?”), or statements about informational needs (“I must get the address where he is now”). Second, the analyst decided which of these requests were open questions, defined as those that projected a substantive answer from the caller, constraining that answer only by topic (e.g., “Where in the stomach is the pain?”). Such questions could be distinguished from ones in which the caller could answer by choosing from a list of option the operator presented (“Is he dry and warm in the skin or pale and clammy?”) or by saying “yes” or “no” (“Are you dry and warm in the skin?”). Note that how the caller actually answered each question was not relevant for this analysis.

#### Acknowledging the caller

Acknowledging the caller was any utterance during which the operator thanked or complimented the caller (“you have been a big help you know”).

#### Expressing empathy to the caller

Analysis focused on identifying when operators displayed empathy to the caller. One challenge was that helping the caller was inherent in the operators’ role; thus, routine, normal helping behaviors could not be considered displays of empathy. Displaying empathy to the caller was therefore defined as (1) deviations from routine (of e.g., questioning, gathering information, advising) to do something that links semantically/affectively to what could reasonably be considered the emotional state of the caller and (2) doing so specifically for *that* caller at *that* moment (i.e., a sympathetic backchannel might fit perfectly in one moment but not another). Operators could fulfil this definition by *affiliating* with the caller’s expressed emotional state (expressing the same), *accommodating* routine responses to match the caller’s expressed emotional state (e.g., doing a backchannel response in a sympathetic tone), or *addressing* possible emotions directly, showing empathy more concretely (e.g., revealing the precise location of an ambulance on its way to the caller).

#### Agreeing with the caller

One of the AMK operators’ primary roles is to assess the distressed person’s medical situation and match it to the available resources. Callers sometimes step into this role; for example, they might diagnose the problem (he’s having a heart attack) and triage (i.e., send an ambulance right away) instead of limiting their contributions to describing the patient’s situation (he has chest pain, is sweaty, and is out of breath). When callers made statements stepping into the operators’ role, they created opportunities for the operator to respond by challenging the statement or the caller’s right to make it. In contrast, the desired response was to avoid saying something negative and instead agree (implicitly or explicitly) and move on with the call. Note that operators did not have to actually agree with the caller or indeed do what the caller wanted; they only had to avoid arguing with or challenging the caller, moving on smoothly while finding a way to fulfill their responsibilities effectively.

Thus, the first step of analysis was to identify all opportunities for agreement in the caller utterances. These were *diagnostic statements* in which the caller defines the problem that the symptoms the caller is experiencing or witnessing (e.g., a heart attack, a gallbladder attack, a stroke), *triaging statements* (e.g., saying that an ambulance was or was not needed), explanations of the *physiological mechanisms* underlying the caller’s immediate situation, or *advising what paramedics or others should do*. The second step was to ascertain whether the operator expressed agreement (explicitly or implicitly) in the next turn.
